# Structural Insights into PROTAC Complex Formation from Analytical Ultracentrifugation and Hydrodynamic Modeling

**DOI:** 10.1063/4.0000810

**Published:** 2025-10-27

**Authors:** Alexander E Yarawsky, Lake N Paul

**Affiliations:** BioAnalysis, LLC, Philadelphia, Pennsylvania, USA

## Abstract

Targeted protein degradation (TPD) has garnered appreciable interest in drug discovery due to its unique mechanism of action – degradation of a target in an event-driven manner, instead of traditional occupancy-driven inhibitor-based therapies. This is achieved by employing mono- or hetero-bifunctional small molecules known as degraders or “PROTACs” to induce the proximity of two proteins: a target protein and an E3 ubiquitin ligase, ultimately resulting in clearance of the target protein by the cell’s inherent degradation machinery. A critical step in this pathway is ternary complex formation (TCF) between the ligase, degrader molecule, and the target protein.

This presentation will demonstrate the implementation of analytical ultracentrifugation (AUC) for obtaining critical attributes of the degrader system. Beyond thermodynamic and kinetic insights, structural information is attainable, including conformational changes upon binary complex formation and characterization of the important ternary complex. Used in conjunction with hydrodynamic modeling, AUC can help confirm the solution-state structure based on predictive or existing structural data.

AUC is a powerful screening tool for degrader-induced ternary complex formation. Once a desirable degrader molecule is identified, a highly rigorous solution-state analysis may be performed to evaluate thermodynamic and structural aspects.

## References

[c1] Yarawsky, A.E., *et al.* A paradigm shift: analytical ultracentrifugation as a multi-attribute platform method in targeted protein degradation. Eur Biophys J (2025). 10.1007/s00249-025-01735-1 39961810

